# Comparison of High-Temperature Compression and Compression-Compression Fatigue Behavior of Magnesium Alloys DieMag422 and AE42

**DOI:** 10.3390/ma13030497

**Published:** 2020-01-21

**Authors:** Mirko Teschke, Alexander Koch, Frank Walther

**Affiliations:** Department of Materials Test Engineering (WPT), TU Dortmund University, Baroper Str. 303, D-44227 Dortmund, Germany; alexander3.koch@tu-dortmund.de (A.K.); frank.walther@tu-dortmund.de (F.W.)

**Keywords:** magnesium, fatigue, compression, rare earth, AE42, DieMag422, tensile test, compression test, ratcheting, high temperature

## Abstract

Due to their high strength-to-weight-ratio, magnesium alloys are very attractive for use in automotive engineering. For application at elevated temperatures, the alloys must be creep-resistant. Therefore, the influence of the operating temperature on the material properties under quasistatic and cyclic load has to be understood. A previous study investigated tensile-tensile fatigue behavior of the magnesium alloys DieMag422 and AE42 at room temperature (RT). The aim of this study was the comparison of both alloys regarding compression, tensile, and compression-compression fatigue behavior. The quasistatic behavior was determined by means of tensile and compression tests, and the tensile-compression asymmetry was analyzed. In temperature increase fatigue tests (TIFT) and constant amplitude tests (CAT), the temperature influence on the cyclic creeping (ratcheting) behavior was investigated, and mechanisms-relevant test temperatures were determined. Furthermore, characteristic fracture mechanisms were evaluated with investigations of the microstructure and the fracture surfaces. The initial material was analyzed in computed tomographic scans and energy dispersive X-ray (EDX) analyses.

## 1. Introduction

Due to their high specific strength, magnesium alloys represent an interesting alternative to materials conventionally used in automotive engineering with regard to reducing vehicle weight. Magnesium alloys have excellent castability and enable thin-walled and complex components to be produced by die casting. With conventional magnesium alloys, the strength decreases and creep deformation increases significantly with increasing temperature. For applications in the powertrain, it is necessary to use alloys that are resistant to creep forming at temperatures above 200 °C. In addition to a thermal load, powertrain applications are subjected to static and cyclic loads in tension and compression direction. The alloy AE42 (Mg-4Al-2SE), which contains rare earth (RE) as alloying elements, is used in the powertrain and has good creep resistance up to 150 °C. Above 150 °C, the creep resistance is significantly reduced so that its use above 175 °C occurring in practice only makes limited sense [1]. In addition, the relatively cheap material magnesium becomes very cost-intensive through the use of expensive RE [2]. For these reasons, alternative creep-resistant magnesium alloys have been developed for many years which do not require RE as alloying elements. A development in this area is the DieMag alloy series, such as DieMag422 (Mg-4Al-2Ba-2Ca/ABaX422). They contain barium and calcium as alloying elements [3].

Dieringa et al. have investigated the static creep behavior of the magnesium alloys DieMag422 and AE42 under tensile and compressive loads [4,5,6]. They have found that the minimum creep rates in tensile and compression creep tests are almost identical at low stresses. At higher stresses, the minimum creep rates deviate from each other and the difference increases with increasing stresses. In previous investigations, the two alloys were tested in continuous load increase tests (LIT) and constant amplitude tests (CAT) under tensile-tensile loadings at room temperature (RT) [7,8,9]. The results show clear differences in the cyclic deformation behavior for both alloys, which affect cyclic creeping (ratcheting) behavior and lifetime at RT. The fatigue strength of AE42 was twice as high as of DieMag422.

The strain accumulation during stress-controlled fatigue tests under the function of non-zero mean stress is known as cyclic ratcheting (cyclic creep) [10,11,12,13]. The hysteresis loop frequently shifts along the strain axis. The dislocation movement is responsible for this. The movement of the dislocations is blocked by obstacles [14]. Increasing temperature leads to thermal activation, which allows a dislocation to overcome one of the obstacles [15]. 

Based on the fact that an asymmetry in the deformation behavior between tensile and compression direction is known from literature [16,17], the compression-compression fatigue behavior of the alloys DieMag422 and AE42 was investigated in this paper. Consequently, the focus of the investigations was on the cyclic creeping (ratcheting) behavior. The investigations were carried out at RT and at relevant elevated temperatures up to 200 °C. In addition, tensile and compression tests were carried out at the same temperatures to investigate the direction-dependent quasistatic deformation behavior.

## 2. Materials and Methods 

The investigated material was produced by the Magnesium Innovation Centre (MagIC), Helmholtz-Zentrum Geesthacht (HZG) (Geesthacht, Germany). The chemical composition of the magnesium alloys DieMag422 and AE42 of the outer area of the cast is given in Table 1. The values were determined by an optical emission spectrometer by HZG. DieMag422 alloy was mixed from a melt containing pure elements, AE42 alloy is commercially available. The material was cast using a permanent mold direct chill casting process. The process is described in detail in Reference [18]. 

**Figure 1:**
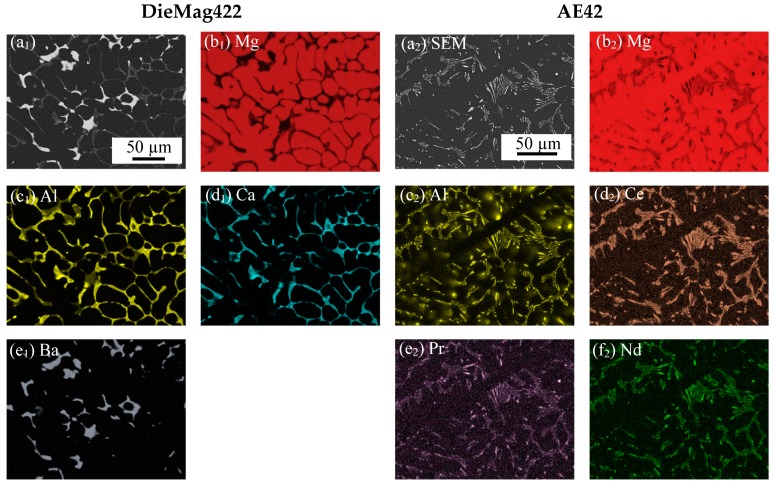
SEM and EDX images of DieMag422 and AE42: (**a**) SEM images; (**b**–**f**) EDX images.

SEM micrographs and energy dispersive X-ray (EDX) analyses for both magnesium alloys were performed using SEM Tescan Mira 3 XMU (Tescan, Brno, Czech Republic) equipped with an EDX detector and the EDAX Team software of EDAX Inc. (Weiterstadt, Germany). The results are given in Figure 1. The EDX analyses and SEM images were used to assign the three phases of DieMag422. The black phase is the α-Mg phase. At the grain boundaries between the α-Mg phase, the grey lamellar Ca- and Al-rich phase Al_2_Ca precipitates [19]. The white phase is Mg_17_Ba_2_ [3]. The two phases are thermally stable up to a test temperature of 535 °C and only transition into the liquid phase at a temperature of 617 °C [3]. The β-phase Mg_17_Al_12_, which is responsible for high creep rates in other casting alloys like AZ91, is suppressed by the formation of Al_2_Ca [20]. For AE42 between the α-Mg phase, precipitations containing Al and RE are formed, which do not form a continuous network between the α-Mg phase. The lamellar precipitates that appear bright in the BSE image are the Al-RE phase, which is responsible for the good creep properties of the AE42 alloy. This prevents the formation of the β-phase Mg_17_Al_12_. Possible precipitate compositions for the Al-RE phase are Al_11_RE_3_, Al_2_RE, and Al_4_RE [21]. The phase Al_11_SE_3_ is only stable up to a temperature of about 150 °C and transforms to Al_2_SE above this temperature [2,21,22]. The released Al forms the γ-phase Mg_17_Al_12_.

**Figure 2:**
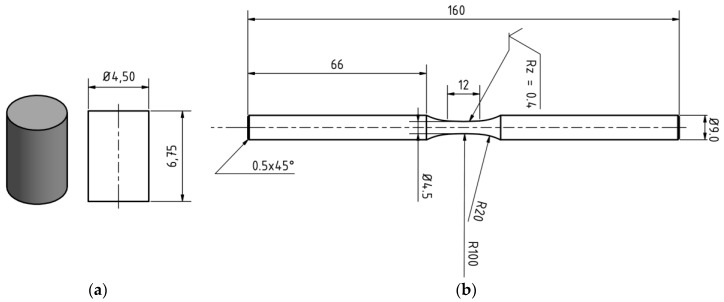
Specimen geometry for mechanical investigations (all dimensions in mm): (**a**) Compression tests and compression-compression fatigue tests; (**b**) tensile tests.

Specimens were extracted from the outer area of the cast. Figure 2a shows the specimen geometry for the compression tests and compression-compression fatigue tests. The specimen geometry for tensile tests is given in Figure 2b. The surface of the specimens was polished using a 3 µm and 1 µm diamond suspension.

**Figure 3:**
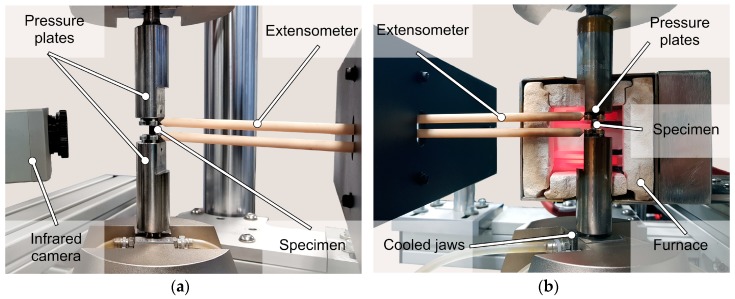
Test setup for quasistatic and compression-compression fatigue tests at room temperature (RT) (**a**) and elevated test temperatures (**b**).

The tensile and compression tests were performed according to standard EN ISO 6892-1/2 (tensile tests) [23,24] and DIN 50106 (compression tests) [25] at room temperature (RT) and at test temperatures of 175 °C and 200 °C. Temperature increase fatigue tests (TIFT) were performed in which the test temperature was increased stepwise to failure under constant cyclic load. All fatigue tests were performed at the servohydraulic testing system Instron 8872 (Instron, Norwood, MA, USA) which has a maximum force of F_max_ = 10 kN. The load-time function was sinusoidal, the load ratio R = 10 and the test frequency f = 10 Hz. In the constant amplitude tests (CAT) at RT, a run-out was defined as 2 × 10^6^ cycles and at elevated temperature as 2 × 10^5^ cycles. The test setup is shown in Figure 3, the applied load in the compression-compression fatigue tests is transmitted to the specimens via pressure plates. The test temperature is generated by a high-temperature furnace, swiveled around specimen setup from both sides. Changes in plastic strain, total mean strain, and temperature were determined as measurement values for material responses. The strain was recorded with the high-temperature extensometer Sandner EXH 10-6A (l_0_ = 10 mm) (Sandner Messtechnik, Biebesheim, Germany); the infrared camera thermoImager TIM 160 (Micro-Epsilon, Ortenburg, Germany) recorded the specimen heating during the fatigue tests at RT.

In order to characterize the inner defect structure, the specimens were analyzed by computed tomography, using Nikon XT H 160 X-ray computed tomography (CT) scanner (Nikon Metrology, Leuven, Belgium). The software VolumeGraphics VGStudioMax 2.2 (Volume Graphics, Heidelberg, Germany) was used to realize the volume reconstructions, as well as the defect analyses. The scanning parameters for the computed tomography scans are given in Table 2. Fractography and microstructural characterization were carried out with an SEM to characterize the fracture mechanisms.

## 3. Results and Discussion

### 3.1. Tensile and Compression Tests

The results of the tensile and compression tests are summarized in Table 3. The characteristic values were determined for DieMag422 and AE42 at RT, 175 °C, and 200 °C. The values were each determined as mean values from three individual tensile/compression tests. The standard deviation is additionally given.

In tensile tests, the 0.2% yield strength (0.2% YS) and ultimate tensile strength (UTS) of DieMag422 for all three test temperatures of RT, 175 °C, and 200 °C are significantly lower compared to AE42. For RT, it is only 42% or 53% of the AE42 value. However, a higher test temperature for DieMag422 alloy does not result in a significant drop in 0.2% YS and UTS. For AE42, increasing the test temperature to 200 °C results in a significant decrease in 0.2% YS (−41%) and UTS (−29%) compared to RT test.

The 0.2% compressive yield strength (0.2% CYS) and ultimate compressive strength (UCS) determined in compression tests are higher for DieMag422 than for AE42 for all three temperatures. At RT, the UCS of AE42 is only 87% of DieMag422. Under compressive load, the UCS and 0.2% CYS for both materials decrease at 175 °C and 200 °C compared to RT. Thus, the 0.2% CYS at 200 °C is only 71% (DieMag422) or 65% (AE42) of the value at RT. The characteristic values determined in compression tests are for both alloys higher than in tensile tests. The 0.2% CYS increases with the increase of the test temperature (from 175 °C to 200 °C) for both investigated magnesium alloys. Máthis et al. relate, that twinning activity probably reached its maximum at 200 °C [26]. Above this temperature, influences by both activity of non-basal slip systems and recrystallization are more predominant [27,28].

**Figure 4:**
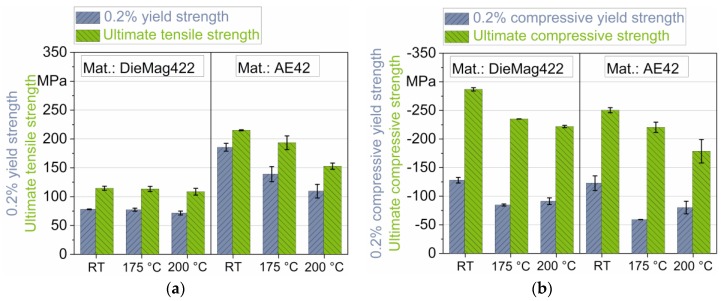
Graphical visualization of the characteristic values from (**a**) tensile and (**b**) compression tests.

Figure 4 shows the same characteristic values as in Table 3 graphically in form of column charts.

Zachariah et al. described the tension-compression asymmetry (TCA) with a ratio factor [29]. According to this, the asymmetry between UTS and UCS, as well as 0.2% YS and 0.2% CYS, is described by the asymmetry ratios TCA_US_ and TCA_YS_, which are listed for pure magnesium [30], DieMag422, and AE42 in Table 4. 

The factors are defined as follows:

(1) $${\mathrm{TCA}}_{\mathrm{US}}=\left|\frac{\mathrm{UTS}}{\mathrm{UCS}}\right|,$$

(2) $${\mathrm{TCA}}_{\mathrm{YS}}=\left|\frac{0.2\%\;\mathrm{YS}}{0.2\%\;\mathrm{CYS}}\right|.$$

The asymmetry between tension and compression direction is much more significant for DieMag422 than for AE42. A significant asymmetry between the 0.2% YS and 0.2% CYS, but also between the UTS and UCS of the same alloy, is often described in the literature for various magnesium alloys [16,30,31]. For pure magnesium and AE42, the 0.2% YS is significantly higher than the 0.2% CYS (TCA_YS_ > 1). This asymmetry was also observed in Reference [16,17,26,32] and decreases with the grain size, thus decreasing twinning. With DieMag422, on the other hand, the opposite is true. The 0.2% YS is significantly lower than the 0.2% CYS (TCA_YS_ < 1). This effect is less significant at increased temperatures. Park et al. [33] observed this behavior on cast AZ31 specimens and explained the tension-compression yield asymmetry by high values of the Schmid factors in the {1012} twin formation in the tensile direction.

The asymmetry between UTS and UCS is described by the factor TCA_US_. For DieMag422, this factor is in the range of pure magnesium, while for AE42, the asymmetry between compression and tensile direction is less pronounced. 

### 3.2. Temperature Increase Fatigue Tests (TIFT)

**Figure 5:**
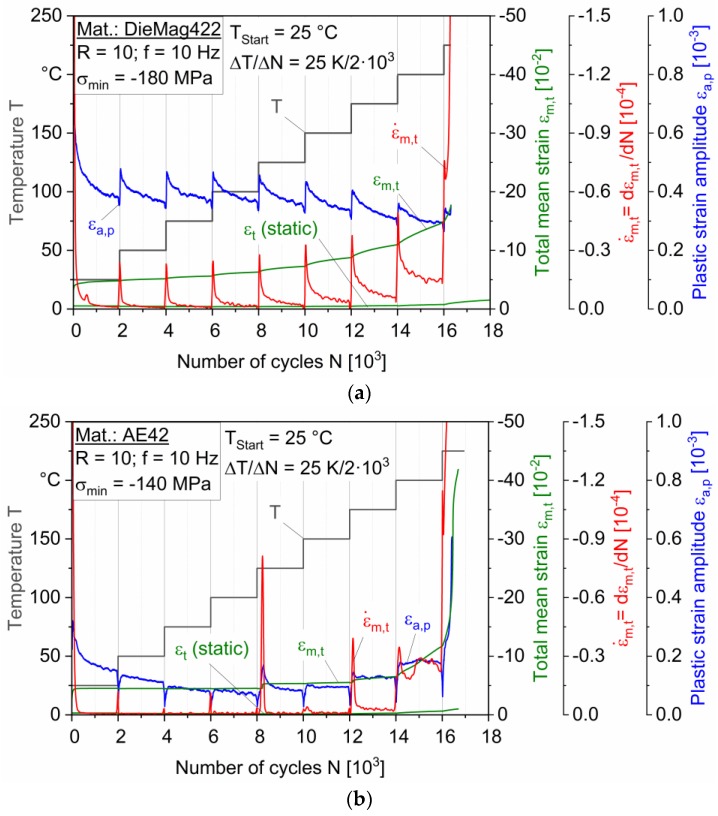
Temperature increase fatigue tests for (**a**) DieMag422; (**b**) AE42.

Figure 5a,b shows the results from TIFT of DieMag422 and AE42, respectively. The test temperature was increased by ΔT = 25 K each 2 × 10^3^ cycles, starting from the start temperature T_start_ = 25 °C. In the tests, the minimum stress was kept constant at σ_min_ = −180 MPa (DieMag422) and at σ_min_ = −140 MPa (AE42). 

For DieMag422 (Figure 5a), the total mean strain indicates an increase over the entire length of the test. Within the temperature steps, the total mean strain increases degressively, recognizable by the sawtooth-like course of the derivation of the mean strain. The derivation of the mean strain describes the gradient of the mean strain. The temperature step of 200 °C is significantly emphasized, as the derivation of the mean strain increases again at the end of the level for the first time. At a temperature of 225 °C, the total mean strain increases steeply and drops at a total mean strain of ε_m,t_ = −17.73 × 10^−2^. The static strain ε_t_ (static) was determined by a separate static creep test with stepwise increasing temperature at the mean stress of σ_m_ = −99 MPa. It is very low compared to the total mean strain ε_m,t_.

The total mean strain of DieMag422 in TIFT increases with each temperature step. This phenomenon is known as cyclic creeping and ratcheting, respectively [15]. Ratcheting becomes more and more significant with increasing temperature. However, the increase is steady. The ratcheting behavior of the alloy DieMag422 can, therefore, be classified as stable since no significant change in the step-related creep rates occurred until failure at the temperature level of 225 °C. From 225 °C, additional pyramidal slipping systems are activated, and the formability of magnesium increases [34,35]. As a result, creep rates increase significantly under static and cyclic loads. 

The plastic strain amplitude of DieMag422 already assumes its maximum value in the first temperature step of 25 °C. Within the temperature steps, the plastic strain amplitude decreases regressively with an increasing number of load cycles. With the start of a new temperature step, the plastic strain amplitude increases significantly and decreases regressively again within the temperature step. This behavior is repeated for the temperature steps until 125 °C. The maxima and minima within the respective temperature levels remain relatively constant in this range. From the temperature step above 150 °C, the qualitative sawtooth pattern continues. However, the maxima at the beginning of each step do not return to the values of the previous step. In further TIFT at higher stress levels, qualitatively comparable trends were observed.

For AE42 (Figure 5b), the plastic strain amplitude shows a regressive decrease until the temperature step of 125 °C is reached. At the beginning of each temperature step, the plastic strain amplitude first drops and then increases again to the plastic strain level of the previous step. Consequently, both alloys differ in their cyclic hardening and softening behavior. At the beginning of a temperature level, DieMag422 hardens and increasingly softens during the temperature step. In contrast, AE42 softens at the beginning of each temperature stage.

The total mean strain of AE42 is constant in the temperature range from 25 °C to 125 °C at about ε_m,t_ = −4.48 × 10^−2^. Between 25 and 100 °C, unlike the analogously tested DieMag422 TIFT, no significant change in the total mean strain is discernible. This can be perceived from the constant derivation of the total mean strain ε_m,t_. At the temperature step of 125 °C, the total mean strain and the plastic strain amplitude increase steeply after about 200 cycles. The increase in the total mean strain is well visible by the peak in the strain rate ${\overset{\text{˙}}{\varepsilon }}_{m,t}$. For DieMag422, no conspicuity can be observed in this temperature range. After the steep rise, the total mean strain returns to an almost constant level. Since this behavior has occurred in all TIFTs performed on AE42, it is apparently a material-related cause. In conventional Mg-Al alloys, the phase Mg_17_Al_12_ softens above 100 °C, but the formation of this phase is suppressed in AE42. Recrystallization processes can also be excluded since magnesium alloys do not recrystallize before 200 °C [36]. 

Up to 175 °C, the total mean strain and the plastic strain amplitude of AE42 are stable, except for the peak at 125 °C. Up to 150 °C, the phase Al_11_RE_3_ is responsible for the good creep properties. Above 150 °C, the Al_11_RE_3_ phase transforms to Al_2_RE, which means the loss of the good creep properties. From a temperature of 225 °C, the activation of the additional pyramidal slipping systems, like DieMag422, leads to a further increase in ductility. In contrast to DieMag422, AE42 can only be described as limited creep resistant regarding static and cyclic creeping. Above 150 °C, cyclical and static creep increases significantly. The results correspond with the observation described by Dieringa et al. [5] that, between 175 °C and 240 °C, DieMag422 shows higher threshold stresses, which means a good creep resistance. The alloy AE42, however, show lower threshold stresses in this temperature range. 

Both magnesium alloys show a clear increase in cyclic ratcheting above 175 °C, as can be seen from the increasing derivation of the total mean strain and the absolute values of total mean strain. For this reason, the test temperatures of 175 °C and 200 °C were selected for further fatigue tests, tensile, and compression tests. 

### 3.3. Constant Amplitude Tests (CAT)

**Figure 6:**
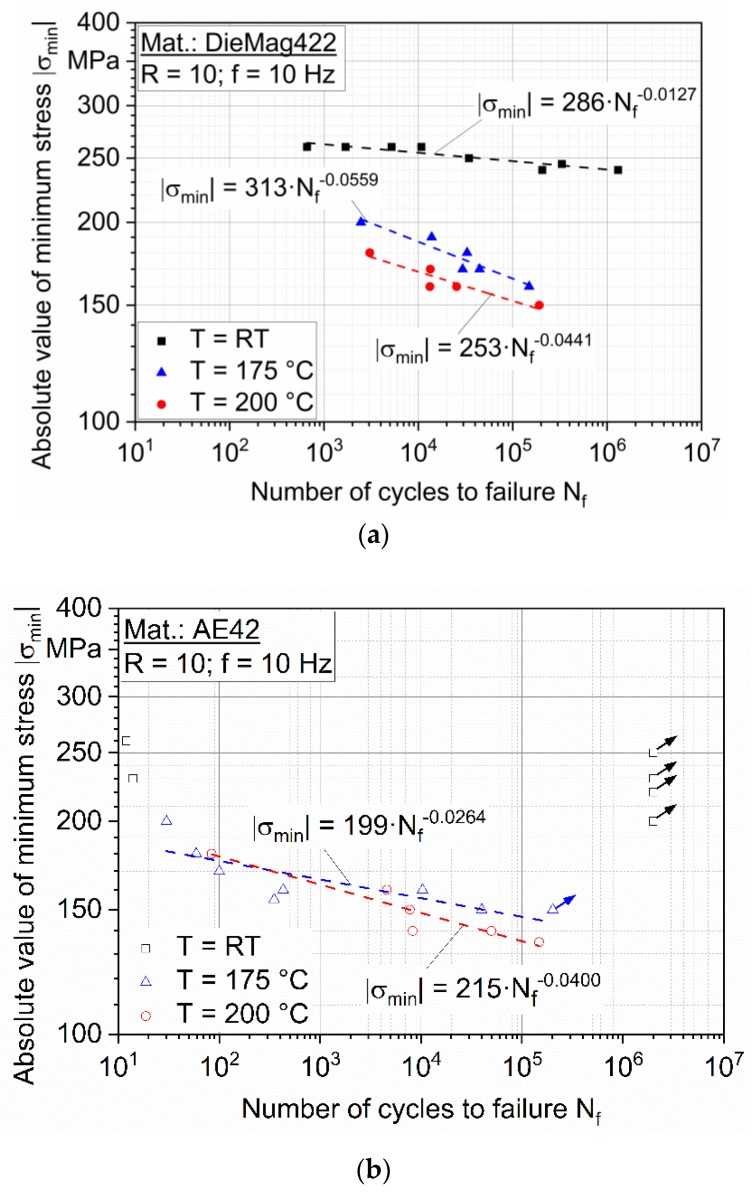
S-N curves for RT, 175 °C, and 200 °C for the alloys (**a**) DieMag422 and (**b**) AE42.

The data points from the CAT of the alloys DieMag422 and AE42 at the three test temperatures RT, 175 °C, and 200 °C were plotted in form of S-N curves for DieMag422 and AE42 in Figure 6a,b, respectively.

For RT, the S-N curve of DieMag422 is significantly flatter than for 175 °C and 200 °C. The fatigue strength at 2 × 10^6^ cycles is estimated to be −238 MPa. The S-N curves at 175 °C and 200 °C decrease significantly more than for the test at RT. For 175 °C, it is at a higher level than for the test at 200 °C. The maximum number of cycles for increased temperature fatigue tests was defined with 2·10^5^ cycles on the basis of the test conditions. Therefore, run-outs occurred at −157 MPa (175°C) and −147 MPa (200°C). For RT, this number of cycles is reached at σ_min_ = −245 MPa. At the test temperature of 175 °C and 200 °C, the minimum stress is thus 64% (175 °C) and 60% (200 °C) of the value at RT. 

For AE42, the S-N curve for 175 °C and 200 °C is at a similar level. The scattering of the S-N curve at 175 °C is high with a coefficient of determination of R^2^ = 0.58. At 200 °C, the coefficient of determination is R^2^ = 0.87. The fatigue strength at N = 2 × 10^5^ cycles is estimated to be −144 MPa for 175 °C and −130 MPa for 200 °C. Similar to the compression tests at 175 °C and 200 °C, the compressive fatigue strength of DieMag422 at increased temperature is higher than that of AE42. For RT, no S-N curve could be created. AE42 shows no metallic fatigue behavior at RT; instead, it is very similar to that of ceramics. It is known that ceramic materials have a very flat S-N curve with a large scattering due to their brittle material behavior [37]. The very flat S-N curves do not permit a technical fatigue limit to be exceeded.

The fatigue strength of DieMag422 at RT in the compression-compression fatigue test is according to amount almost 400% of the tensile-tensile fatigue strength determined by Klein et al. [7]. Conversely, the tensile-tensile fatigue strength of AE42, similar to the tensile test, is higher than that of DieMag422. This result is an analog to the creep tests of Dieringa et al. [1].

### 3.4. Microstructural Characterization

**Figure 7:**
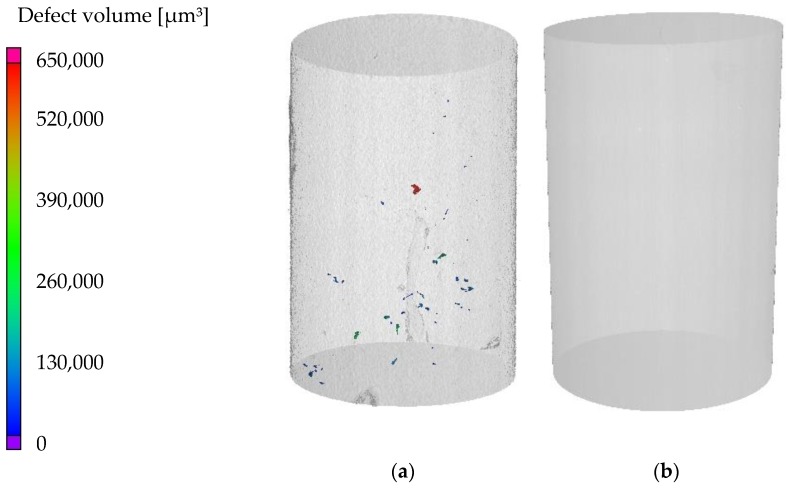
Volume reconstruction with detected defects in (**a**) DieMag422 specimen and (**b**) AE42 specimen.

Figure 7a,b shows the volume reconstructions of a compression fatigue specimen of DieMag422 and AE42, respectively, in the untested state. In the DieMag422 specimen, the pores are irregularly distributed. 

**Figure 8:**
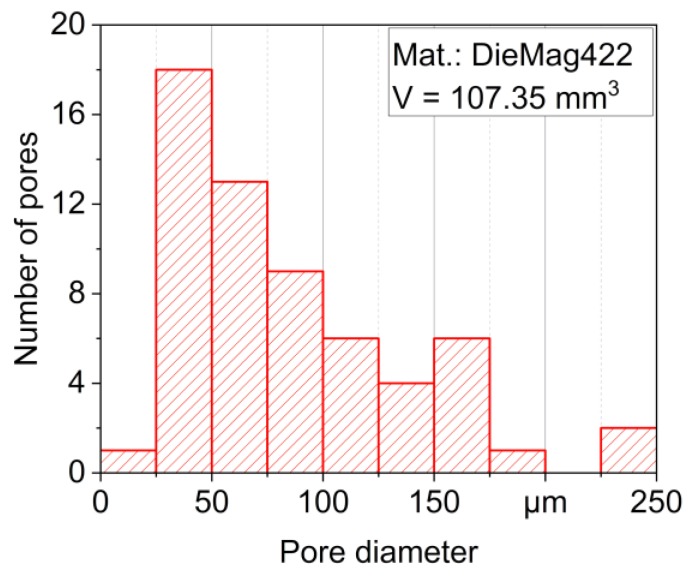
Representation of the pore distribution in a DieMag422 fatigue specimen by histogram.

The histogram in Figure 8 shows that most of the pores have a diameter between 25 and 175 µm. A few pores are larger, the largest having a diameter of more than 290 µm. Conversely, only one pore with a diameter of 18.45 µm could be detected in the AE42 specimen. The exemplarily scanned AE42 specimen is, therefore, almost pore-free. Although both materials were produced with the permanent mold direct chill casting process, known for its particularly high density, clear differences can be observed. Although DieMag422 has more pores, the DieMag422 specimens have a significantly lower scattering in the S-N diagram compared to the AE42 specimens.

**Figure 9:**
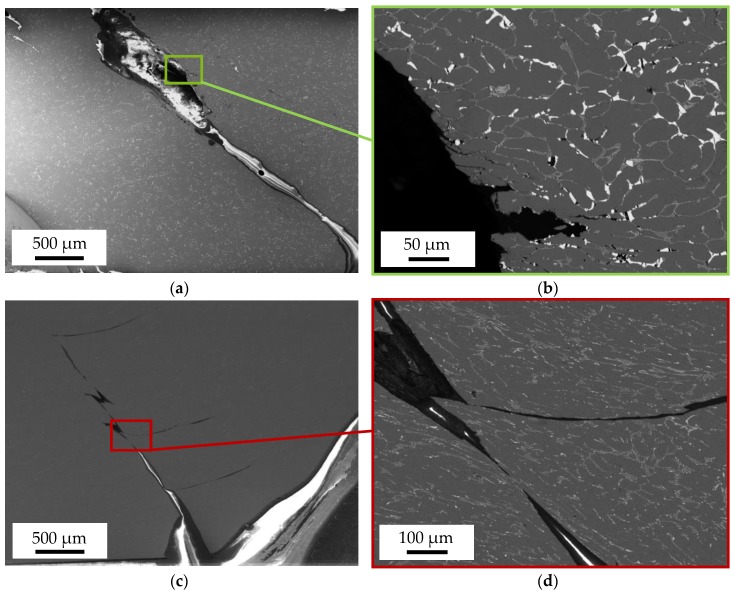
Longitudinal section of the fracture surface of a DieMag422 (**a** + **b**) and AE42 (**c** + **d**) specimen tested at constant amplitude testing at 200 °C.

Figure 9 shows the longitudinal section of the fracture surface of a DieMag422 (Figure 9a,b) and AE42 (Figure 9c,d) specimen taken with the BSE detector of the SEM and tested at a test temperature of 200 °C. For DieMag422, the minimum stress in the CAT is σ_min_ = −180 MPa. Fracture occurred at ~45° with respect to the compression test axis (Figure 9a), which represents the direction of maximum shear stress. As can be seen in the close-up (Figure 9b), the fracture grows intercrystalline in the middle of the specimen. In the edge area, the crack grows transcrystalline. The single grains indicate the entire specimen has been compressed. Parallel to the crack at an angle of 45°, further small transcrystalline secondary cracks can be observed, which could have been caused by the union of pores of various sizes.

At the more brittle AE42 specimen, which was tested at σ_min_ = −180 MPa in a CAT, fracture equally occurred at ~45° with respect to the compression test axis (Figure 9c). However, this crack is transcrystalline. Further large transcrystalline secondary cracks running at an angle of 90° to the loading direction can be observed (Figure 9d). Due to the strong cyclic ratcheting, especially at increased test temperatures, high compression occurs under cyclic load. No pores are visible.

**Figure 10:**
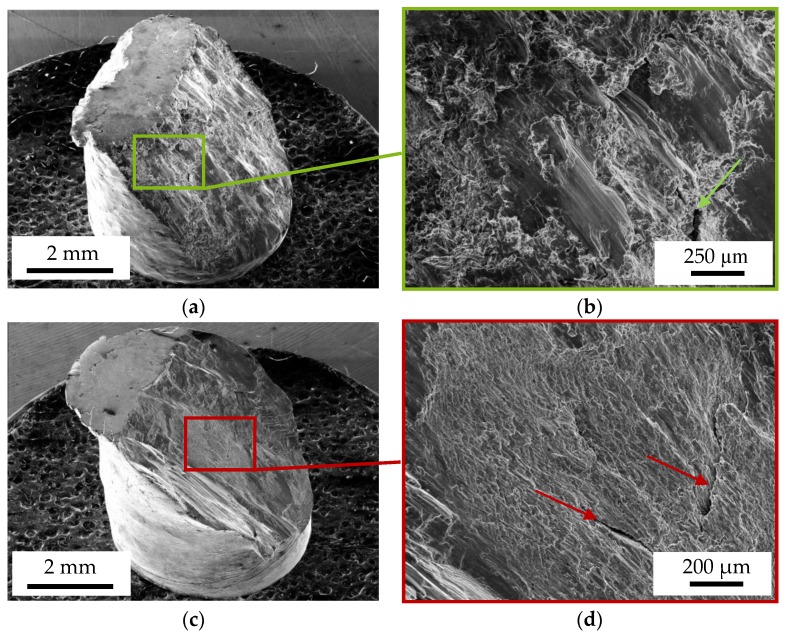
Fracture surface of a DieMag422 (**a** + **b**) and AE42 (**c** + **d**) specimen tested at constant amplitude tests (CAT).

Figure 10 shows the fracture surfaces of the DieMag422 (Figure 10a,b) and AE42 (Figure 10c,d) specimens taken with the SE detector of the SEM. For the images, the specimen table of the SEM was tilted by 45°. The specimens are each cracked at an angle of 45° to the compression test axis. 

For DieMag422, plastic deformations can be observed on the lateral surface (Figure 10a). The fracture surface shows various structures that can be divided into two areas. In addition to the flat fracture surface areas where the two fracture surfaces have slipped against each other, a fracture surface with a dimpled structure can be seen in the specimens that failed in CAT. Inside the fracture surface, single cracks marked with an arrow are visible (Figure 10b), which were already identified as transcrystalline cracks in the longitudinal section in Figure 9b. DieMag422 fracture surfaces show no differences in the fractographic images depending on the test temperature.

For AE42, unlike DieMag422, no plastic deformations on the lateral surface can be detected. As in the compression tests, two different specimen areas can also be identified here. In addition to the flat fracture surface areas, which were created by the two fracture surfaces sliding on each other, a dimpled structure is visible, which can equally be seen on the fracture surfaces of the AE42 compression test. Figure 10d also shows some secondary cracks marked with arrows, which were already identified as transcrystalline cracks in the longitudinal section in Figure 9d.

The cracking behavior, under an angle of 45° to the compression test axis, was also observed in Reference [30] on pure magnesium, in Reference [38] on the alloys Mg4Gd and Mg6Ag, and in Reference [39] on a magnesium-zinc-gadolinium alloy that contains RE. The fracture surfaces from the compression tests, as well as the compression-compression fatigue tests, show similar structures. Therefore, it can be assumed that failure under cyclic load occurs only in the last cycles. However, further investigations to get a precise understatement of the damage development are necessary. Since DieMag422 shows ductile material behavior, the dimpled structures are very prominent and typical for shear fractures. The less ductile material AE42, on the other hand, has significantly flatter dimpled structures. The wavy pattern of a compression test specimen described in Reference [39] by Seetharaman et al. on RE-containing magnesium-zinc-gadolinium alloys resembles the wavy structures which can be observed particularly pronounced on the AE42 specimens. These structures are typical for shear mode fracture behavior of magnesium alloys.

## 4. Conclusions

In this paper, a newly developed test rig for high-temperature quasistatic tensile and compression tests, as well as compression-compression fatigue tests, was presented. The conventional RE-containing magnesium alloy AE42 and the magnesium alloy DieMag422 produced without RE were comparatively investigated regarding its mechanical high-temperature behavior.

In addition to the method of the load increase test, a new method, the temperature increase fatigue test, proved to be suitable for the identification of critical test temperatures in fatigue tests since relevant test temperatures can be identified using only one specimen. In addition, information about the temperature-dependent cyclic hardening and softening, as well as cyclic creeping (ratcheting) behavior, can already be gained. Using this new test method, the number of further required tests, such as the constant amplitude test, can be significantly reduced.

In quasistatic tensile and compression tests, a distinct asymmetry of the ultimate strength and 0.2% yield strength between tension and compression direction for three test temperatures (RT, 175 °C, and 200 °C) was determined and could be described with asymmetry ratios. The ultimate compressive strength was more than 100% (DieMag422) or 12% (AE42) higher than the ultimate tensile strength, although the asymmetry was more pronounced for DieMag422. 

In compression-compression fatigue tests increasing temperatures lead to decreasing compression-compression fatigue strength for both alloys. The compression-compression fatigue strength of DieMag422 at increased temperature is higher than that of AE42. Compared to tensile-tensile fatigue tests from other studies, the compression-compression fatigue strength of DieMag422 at RT was found to be higher. For AE42, a reversed fatigue behavior could be observed.

With the help of EDX-mappings, the microstructure of the alloys was analyzed. Investigations of the fracture surface with fractographical images and longitudinal sections showed that the fracture occurred at ~45° with respect to the compression test axis at quasistatic compression tests and compression-compression tests. For DieMag422, a compound of intercrystalline and transcrystalline crack propagation was observed, while for AE42, only transcrystalline crack propagation was observed.

## 5. Outlook

In order to understand the reason for the asymmetric deformation behavior under tensile and compressive load between the alloys DieMag422 and AE42 in more detail, EBSD investigations should be carried out on the broken tensile and compression test specimens. In addition to the tensile-tensile fatigue investigations at RT [7,9], investigations at elevated temperature should also be carried out. In order to get a precise understanding of the damage development, such as the location of the initial crack and the crack propagation rate, further investigations will be necessary in the future. For this reason, intermittent fatigue tests in combination with computed tomography scans are recommended, as done in Reference [40]. Due to the asymmetry found in the tensile and compression tests, as well as the fatigue tests of both alloys, additional fatigue tests with a stress ratio of R = −1 should be carried out. Due to the different Young’s modulus and yield strength in the respective half cycle (tension/compression), cyclic ratcheting is to be expected. Similar tests were performed in Reference [41,42].

## Figures and Tables

**Table 1:** Chemical composition of magnesium alloys DieMag422 and AE42 (wt.%).

Alloy	Mg	Al	Ba	Ca	Ce	Nd	Pr	Y	Others
DieMag422	Bal.	4.82	1.54	1.88	-	-	-	-	<0.001
AE42	Bal.	4.34	-	-	1.40	0.46	0.1	0.011	<0.001

**Table 2:** Scanning parameters for the computed tomography scans (μ-CT).

Material	Beam Energy	Beam Current	Power	Effective Pixel Size	Exposure Rates	
DieMag422, AE42	86 kV	41.9 µA	3.6 W	5 µm	1.42 s	0.707 fps

**Table 3:** Characteristic values from tensile and compression tests for DieMag422 and AE42 at the test temperatures of room temperature (RT), 175 °C, and 200 °C.

Alloy	T	0.2% YS ^1^ (MPa)	UTS ^2^ (MPa)	0.2% CYS ^3^ (MPa)	UCS ^4^ (MPa)
DieMag422	RT	78.1 ± 0.6	114.6 ± 3.5	−128.0 ± 4.9	−286.8 ± 2.9
	175 °C	77.4 ± 2.6	113.3 ± 4.5	−84.6 ± 1.8	−235.1 ± 0.3
	200 °C	71.6 ± 3.4	108.6 ± 5.8	−91.2 ± 5.9	−221.9 ± 1.9
AE42	RT	185.6 ± 6.8	215.1 ± 0.9	−122.8 ± 12.8	−250.4 ± 4.4
	175 °C	139.1 ± 13.1	193.5 ± 11.8	−59.1 ± 0.3	−220.5 ± 9.0
	200 °C	109.5 ± 11.8	152.8 ± 5.4	−80.2 ± 11.0	−178.6 ± 20.6

^1^ 0.2% yield strength; ^2^ Ultimate tensile strength; ^3^ 0.2% compressive yield strength; ^4^ Ultimate compressive strength.

**Table 4:** Comparison of the asymmetry ratios TCA_YS_ and TCA_US_ for pure magnesium, DieMag422, and AE42. TCA = tension-compression asymmetry.

Alloy	T	TCA_YS_	TCA_US_
Pure Mg [30]	RT	1.61	0.47
DieMag422	RT	0.61	0.40
	175 °C	0.91	0.48
	200 °C	0.79	0.49
AE42	RT	1.51	0.86
	175 °C	2.35	0.88
	200 °C	1.36	0.86
